# Impact of Different Oxygen Supply Methods on the Healing of Corneal Epithelial Wound and the Level of Acetylcholine

**DOI:** 10.1155/2021/4737479

**Published:** 2021-11-20

**Authors:** Shanshan Li, Gang Ding, Yuqin Sun, Chenming Zhang

**Affiliations:** Jinan Second People's Hospital, Jinan 250001, China

## Abstract

**Purpose:**

To investigate the impact of different oxygen supply methods on corneal epithelial wound healing and acetylcholine level during wound healing.

**Methods:**

We randomly divided 75 rabbits into three groups: A, B, and C, with 25 rabbits in each group. The central corneal epithelium was removed from all eyes of the rabbits using a 5 mm trephine. Group A rabbits were given low flow oxygen (3 L/min; concentration: 33%) for 2 h per day through goggles. Group B rabbits were given low flow oxygen (3 L/min; concentration: 33%) for 2 h per day via oxygen masks for inhalation. Group C rabbits healed naturally. The area of healed corneal epithelium and acetylcholine content in corneal epithelium were determined at 12 h, 24 h, and 36 h after injury.

**Results:**

At 12 h, 24 h, and 36 h after injury, the healing area of corneal epithelium in the three groups was in the order group A > group B > group C (*P* < 0.05). At all timepoints, the acetylcholine level in corneal epithelium was in the order of group A > group B > group C (*P* < 0.05). In all three groups, the acetylcholine content in corneal epithelium showed the order 12 h > 24 h > 36 h (*P* < 0.05). There was a correlation between acetylcholine expression and the area of unhealed corneal epithelium, and the correlation coefficients of groups A, B, and C were 0.80, 0.83, and 0.85 respectively.

**Conclusion:**

Increasing oxygen concentration through inhalation or via goggles can promote corneal epithelial wound healing, but increasing local oxygen concentration of the eye showed a better effect. Acetylcholine may play an important role in the early process of corneal epithelial wound healing.

## 1. Introduction

Corneal epithelium is the outermost layer of the cornea, which protects the cornea against the infection caused by harmful environmental factors [1, 2]. Corneal epithelial erosion is one of the most common clinical problems in ophthalmology. Continuous loss of epithelial integrity can lead to infection, stromal edema, or corneal ulcer, and corneal wound healing defects can cause ocular surface damage [3, 4]. During corneal epithelial wound healing, normal corneal nerves release certain levels of neurotransmitters, which play an important role in the active transport of corneal ions, epithelial mitosis, and corneal wound healing [5]. Therefore, increasingly understanding the cell and molecular mechanisms of corneal epithelial regeneration may facilitate the prevention and treatment of persistent corneal epithelial defects. Acetylcholine is an important neurotransmitter in the corneal epithelium. It is produced by almost all types of living cells, and its concentration in corneal epithelium is very high, which is higher than that in other nerve tissues [6, 7]. Recently, muscarinic receptor and nicotinic receptor have been discovered in human and mouse corneal epithelium [8], which supports the view that corneal epithelium may be a potential target of acetylcholine receptor in some neural information transmission pathway [9].

Oxygen therapy has been used in clinics for many years [10]. In clinical ophthalmology, oxygen is often used in the treatment of corneal chemical burn and scleritis as hyperbaric oxygen therapy [11–13]. In this study, we investigated the impact of increasing oxygen supply to the eye either via oxygen goggles or via oxygen mask for inhalation on corneal epithelial wound healing in a rabbit corneal epithelial injury model.

## 2. Materials and Methods

### 2.1. Materials

Total of 75 healthy New Zealand white rabbits (150 eyes) weighing 2–2.5 kg (2.256 ± 0.244 kg, *P* > 0.05) were used in the study. The study was carried out at the Institute of Ophthalmology, Jinan Second People's Hospital. The rabbits were intramuscular injected with anesthetic solution containing ketamine hydrochloride 50 mg kg^−1^, chlorpromazine hydrochloride 10 mg kg^−1^, and atropine sulfate 0.015 mg kg^−1^. After general anesthesia, the eyes of the rabbits were washed with sterile normal saline and routinely disinfected. Sterile towels were draped around the surgical area. The rabbits were sacrificed by air embolism via the ear vein, and the corpses were properly stored at −20°C. Other supplies included low flow oxygen goggles that can be connected to oxygen trachea, oxygen mask, and acetylcholine (ACh) test kit.

### 2.2. Methods

#### 2.2.1. Establishment of the Corneal Epithelial Injury Rabbit Model

The central corneal epithelium of all rabbits was stripped using a 5 mm trephine. Group A rabbits were given oxygen goggles every day, and low flow oxygen (3 L/min, concentration: 33%) was given through the goggles once a day for 2 h. Group B rabbits were given low flow oxygen (3 L/min, concentration: 33%) via oxygen masks for inhalation once a day for 2 h. Group C rabbits healed naturally without any treatment. Levofloxacin eye drop was given to all rabbits four times a day every day.

#### 2.2.2. The Area of Healed Corneal Epithelium at Each Timepoint

At 12 h, 24 h, 36 h, 48 h, and 60 h after corneal epithelial curettage, corneal fluorescence staining was performed, and images of stained samples were taken under the slit lamp with the ruler as the reference, and the area of unhealed corneal epithelium in each group as indicated by fluorescence staining was assessed using ImageJ.

#### 2.2.3. Determination of Acetylcholine Content in Corneal Epithelium

At 12 h, 24 h, and 36 h after the removal of corneal epithelium, five rabbits were randomly selected in each group and sacrificed by air embolism. Ten corneas were collected from the five rabbits in each group, and each cornea was cut into two parts with the same size along the diameter. One of the two halves of the cornea was placed in 2.5% glutaraldehyde phosphate buffer within 30 s after cutting and stored at 4°C. The content of acetylcholine in corneal epithelium was determined by ELISA. The specific procedure of acetylcholine determination was as follows: the standard, reagents, and samples were prepared before the test. After adding 50 *μ*L sample (containing the standard and sample) to the reaction plate, 50 *μ*L test solution A was immediately added to the sample and the plate was incubated at 37°C for 1 h. The plate was tumble dried and washed three times. After adding 100 *μ*L of test solution B, the plate was incubated at 37°C for 30 min. The plate was washed five times. After adding 90 *μ*L TMB substrate, the plate was incubated at 37°C for 10–20 min. Finally, 50 *μ*L of the termination solution was added to the reaction solution, and the plate was read immediately at 450 nm wavelength.

### 2.3. Statistical Analysis

The area of fluorescence-stained corneal epithelium was analyzed using ImageJ, and the area of healed corneal epithelial in groups A, B, and C at 12 h, 24 h, and 36 h after injury was calculated and analyzed using the univariate ANOVA test. The content of acetylcholine in each group at 12 h, 24 h, and 36 h after corneal epithelial removal was analyzed using the univariate ANOVA test. The correlation between the area of unhealed corneal epithelium and acetylcholine content was determined using linear correlation analysis. All data were recorded as the mean ± standard deviation, and *P* < 0.05 was considered statistically significant. SPSS 10.0 software was used for statistical analysis.

## 3. Results

### 3.1. Corneal Epithelial Wound Healing after Injury

**Figure 1:**
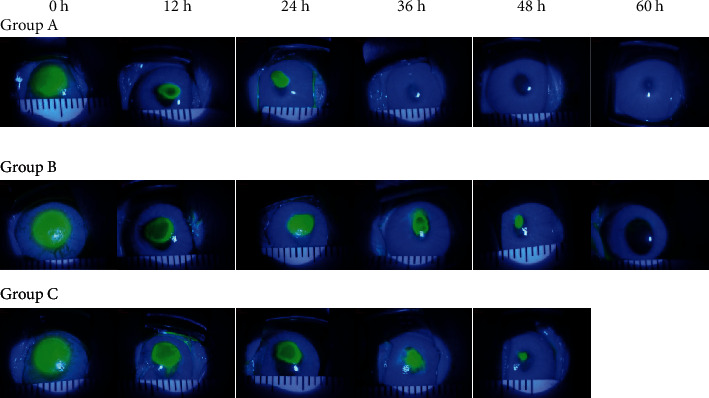
The fluorescence staining of corneal epithelium in each group at different time points shows that in groups A, B, and C, the area of fluorescence staining on the cornea was the same at 0 h, when the experiment was initiated, but at 12 h and 24 h after intervention, the area of fluorescence staining in group A was significantly smaller than that of group B and group C. At 36 h, filamentous fluorescence staining was only seen in the center of the cornea, and the surrounding cornea tissue was transparent. At 48 h, no corneal fluorescence staining was found, indicating that the corneal epithelium was completely healed. In group B, the area of corneal fluorescence staining was larger than that of group A but smaller than that of group C at all time points except at 0 h. In group C, the area of fluorescent staining was the largest among all three groups at all time points. After 72 h of intervention, filamentous fluorescence staining still could be seen, indicating that corneal epithelium was rough and not completely healed.

At 0 h, 12 h, 24 h, 36 h, 48 h, and 60 h after corneal epithelium was scraped, the fluorescence staining of corneal epithelium under slit lamp is shown in Figure 1. The results showed that the staining area of group A at each time point was significantly smaller than that of group B, and the staining area of group B was smaller than that of group C. It indicated that at the same time point, the healing area of group A was the largest, followed by group B and group C.

The area of fluorescence-stained corneal epithelium was analyzed using ImageJ software to evaluate the area of unhealed corneal epithelium at 12 h, 24 h, and 36 h after injury (Table 1).

At 12 h, 24 h, and 36 h after corneal epithelial removal, the area of unhealed corneal epithelial at each time point was group A < group B < group C, with statistically significant differences (*P* < 0.05). The unhealed area in group C was the largest, followed by group B, and the smallest in group A. There was a significant difference between any of the two groups (*P* < 0.017).

The content of acetylcholine (pg/mL) in corneal epithelium was measured at 12 h, 24 h, and 36 h after injury (Table 2).

At 12 h, 24 h, and 36 h after corneal epithelium removal, the content of acetylcholine in the three groups followed the same trend: acetylcholine content was the highest in group A (goggles group), followed by group B (oxygen mask for inhalation group), and group C (natural healing group) had the lowest acetylcholine content (group A > group B > group C, *P* < 0.05). Comparing the content of acetylcholine at 12 h, 24 h, and 36 h after corneal epithelium injury, we found that the acetylcholine content in each group was in the order 12 h > 24 h > 36 h (Table 2, *P* < 0.05), and there were statistically significant differences among the three groups (*P* < 0.017).

### 3.2. The Correlation between the Area of Unhealed Corneal Epithelium and Acetylcholine Content in Different Groups

Group A showed correlation coefficient (*r*) = 0.80202036, *t* = 5.2004, *P* ≤ 0.001. Group B showed correlation coefficient (*r*) = 0.83090169, *t* = 6.6782, *P* ≤ 0.001. Group C showed correlation coefficient (*r*) = 0.85036007, *t* = 7.9169, *P* ≤ 0.001. Our results showed that the area of unhealed corneal epithelium had positive correlation with the content of acetylcholine in groups A, B, and C rabbits.

## 4. Discussion

The cornea is the transparent fibrous membrane that comprises the outer layer of the eyeball and forms the first barrier of the eye. Because of the tight connection between epithelial cells, corneal epithelium has the function of semipermeable membrane and provides a barrier between the external environment and the corneal matrix [14]. Once corneal epithelium is damaged, it will immediately respond to the damage, heal the wound in the way of lamellar migration, cover the defect area, and reconstruct its barrier function [15]. The damaged corneal epithelial cells and the secondary damaged corneal stromal cells will release cytokines and chemokines, which act on corneal stromal cells and cause a series of pathophysiological changes in these cells. The crucial events in this process are cell migration and proliferation, which is driven by the coordinated release of growth factors to the injured site. In the injured cornea, epithelial cells play a central role; they are not only the critical cell type to repair the cornea but also the source of some growth factors. Like in other tissues, many growth factors play important roles in regulating corneal epithelial function and wound healing [16]. In this study, we mainly focused on one of such factors: acetylcholine for its role in corneal epithelial wound healing.

In clinical practice, oxygen is often used as hyperbaric oxygen therapy to help wound healing and as heat burn treatment [17]. However, there are only a few reports regarding its use in ophthalmology [11–13]. Although some scholars believe that the beneficial effect of oxygen in tissue healing depends on oxygen partial pressure, the higher the oxygen partial pressure, the better the injured tissue heals [18]; in this study, we did not provide conventional hyperbaric oxygen to the rabbits to facilitate wound healing. Instead, we provided oxygen to the rabbits in two ways: local oxygen supply through goggles and systemic oxygen supply through oxygen masks for inhalation, mainly because these two oxygen delivery methods are more in line with clinical oxygen supply methods. In our study, we found that the two oxygen supply methods can significantly accelerate corneal epithelial wound healing after injury (Figure 1). The healing of corneal epithelium was the fastest in the group with local oxygen supply via goggles (Table 1), which indicates that oxygen can promote the recovery of corneal epithelium, and the wound healing promotion effect is the most significant when oxygen is given locally to the eye. It is known that oxygen is capable of favorably influencing a number of cytokines and growth factors that play an important role in wound healing [10]. However, there is no clinical report regarding the impact of oxygen on acetylcholine expression. In this study, we established a corneal epithelial injury rabbit model and provided oxygen supply to the rabbits in two different ways. We found that oxygen may promote the production of acetylcholine, and the content of acetylcholine in the cornea was the highest when oxygen was given locally via goggles, showing that the local oxygen supply method has the most significant acetylcholine production promotion effect (Table 2). Our results suggested that oxygen can increase the production of acetylcholine, which may be an important mechanism through which oxygen promotes corneal epithelial wound healing.

In the cornea, ACh largely resides within the epithelial cells, but not neural cells [19]. Moreover, some classic studies have shown that mammalian corneal epithelium contains high concentration of endogenous acetylcholine (ACh) and high levels of choline acetyl transferase (ChAT) and acetylcholinesterase (AChE) activities [20–23]. At present, two basic types of membrane receptors-muscarinic receptors (mAChRs) and nicotinic acetylcholine receptors (nAChRs) are known to mediate the effects of acetylcholine on cells [24, 25]. Studies have shown that cholinergic receptor agonist acetylcholine (ACh) can increase the tissue level of endogenous acetylcholine and accelerate corneal epithelial regeneration by activating mAChRs and nAChRs, acetylcholinesterase (AChE) muscarinic antibody, nicotinic receptor agonist carbachol chloride (CCH), and irreversible AChE inhibitor echothiophate [26]. The action of acetylcholine is not only from nerve to nerve but also from nerve to other nonneural cells such as muscle cells and glands. ACh plays an important role in the control of mitotic rate in epithelial cells and corneal epithelial wound healing [27, 28]. Therefore, any interference on the balance of Ach-AChE, no matter being induced physically or chemically, may affect corneal wound healing, indicating that acetylcholine is important in promoting corneal epithelial healing. In this study, we investigated the impact of different oxygen delivery methods on the level of acetylcholine in cornea. We found that at 12 h, 24 h, and 36 h after injury, increasing oxygen supply to the eye via oxygen mask for inhalation or via goggles can promote the expression of acetylcholine in cornea, and the acetylcholine expression promotion effect was in the order group A > group B > group C (Table 2). The expression of acetylcholine was the highest in local oxygen supply via the goggles group. Based on these results, we concluded that oxygen may promote the expression of acetylcholine in cornea, and local oxygen supply has the most significant effect. Regarding corneal epithelial wound healing at different time points, we found that in all of the three groups, the expression of acetylcholine was the highest at 12 h after injury, followed by 24 h and 36 h (12 h > 24 h > 36 h) (Table 2). Therefore, we speculated that acetylcholine may be crucial for the early repair of corneal epithelium after injury.

Clinical data have shown that oxygen enhances the release of cytokines and growth factors to promote tissue healing [10], while acetylcholine in corneal epithelium promotes corneal epithelial wound healing through a series of synaptic transmission and transmembrane transport [29]. In our study, we calculated the area of healed corneal epithelium and the content of acetylcholine in the cornea under three healing conditions and analyzed the correlation between the area of healed corneal epithelium and the acetylcholine expression in the cornea. The results showed that there was a positive correlation between the area of unhealed corneal epithelium and the acetylcholine content. However, the causal relationship between them still needs to be determined. This result further confirmed that acetylcholine may play an important role in corneal epithelial wound healing. Due to the small sample size and the relatively fixed oxygen concentration, future studies with expanded sample size and different concentrations of oxygen are necessary to further verify the effect of oxygen on corneal epithelial wound healing, so as to provide practical guide for the clinical application of oxygen in ophthalmology.

## 5. Conclusions

Corneal wound healing is a complex process that involves multiple factors such as cells, stroma, and secretory factors to repair the damaged tissue. Our study showed that increasing oxygen supply, no matter through goggles or oxygen mask for inhalation, can promote the corneal epithelial wound healing after injury, and the local oxygen supply through goggles has the best beneficial effect. Acetylcholine may play an important role in the early repair of corneal epithelium following injury. Increasing local oxygen supply can promote the expression of acetylcholine in corneal epithelium, help maintain the stable state of normal corneal epithelium, and facilitate wound healing. This mechanism may provide a new approach for the clinical treatment of corneal epithelial diseases.

## Figures and Tables

**Table 1:** The area of unhealed corneal epithelium in each group at 12 h, 24 h, and 36 h after corneal epithelium removal.

	12 h	24 h	36 h	T (12 h vs. 24 h)	P (12 h vs. 24 h	T (12 h vs. 36 h)	P (12 h vs. 36 h)	T (24 h vs. 36 h)	P (24 h vs. 36 h)
A (*n* = 10)	11.0850 ± 0.6210	4.9509 ± 3.7226	0.8805 ± 1.3312	5.1398	0.0001	21.9681	0.0001	3.2558	0.0044
B (*n* = 10)	22.8885 ± 0.6347	9.2520 ± 1.2867	4.2563 ± 3.2244	30.0526	0.0001	17.9292	0.0001	4.5505	0.0002
C (*n* = 10)	34.9433 ± 3.5433	18.3283 ± 0.7466	8.2720 ± 3.1222	14.5097	0.0001	17.8592	0.0001	9.9061	0.0001
F	287.95	24.78	16.87						
P	0.0001	0.0001	0.0001						

**Table 2:** Acetylcholine content in corneal epithelium of each group at 12 h, 24 h, and 36 h after corneal epithelium removal.

	12 h	24 h	36 h	T (12 h vs. 24 h)	P (12 h vs. 24 h)	T (12 h vs. 36 h)	P (12 h vs. 36 h)	T (24 h vs. 36 h)	P (24 h vs. 36 h)
A (*n* = 10)	20.3891 ± 5.312	11.7864 ± 0.4752	6.9132 ± 2.3250	5.1009	0.0001	7.3492	0.0001	6.4939	0.0001
B (*n* = 10)	12.493 ± 4.2596	7.2531 ± 1.3623	3.9121 ± 2.2160	3.7052	0.0016	5.6513	0.0001	4.0616	0.0007
C (*n* = 10)	7.9417 ± 2.1845	2.7761 ± 0.5367	0.8905 ± 1.2670	7.2618	0.0001	8.8297	0.0001	4.3335	0.0004
F	23.27	9.04	15.48						
P	0.0001	0.0010	0.0001						

## Data Availability

The data generated or analyzed during this study are available from the corresponding author upon reasonable request.
